# Is reviewing trial protocols on clinicaltrials.gov a feasible method of compiling a long-list for a core outcome set?

**DOI:** 10.1186/1745-6215-16-S3-P6

**Published:** 2015-11-24

**Authors:** Michael Fabricius, Robert Pickard, Elaine McColl

**Affiliations:** 1 Northern Institute for Cancer Research, Newcastle University, Newcastle upon Tyne, UK; 2 Institute of Cellular Medicine, Newcastle University, Newcastle upon Tyne, UK; 3 Institute of Health and Society, Newcastle University, Newcastle upon Tyne, UK

## Background

There is a lag of several years between the design and registration of a trial, and the publication of results, therefore the outcome measures extracted from a SR may not represent current practice. Furthermore, carrying out a SR is a laborious and time-consuming process. We sought to determine whether a review of trial registry records would be an efficient alternative to a SR.

## Methods

We carried out a SR of advanced prostate cancer (PC) trials published over the period 2008-2013 (reported separately) and then reviewed the corresponding trials registry entries for the studies included in the SR. Clinicaltrials.gov NCT registration numbers were extracted from the papers where available. Where an NCT number was not available, the registry was searched for the study. A table of primary and secondary outcome measures was compiled and compared with the published outcomes.

## Results

NCT numbers were available for 37/47 of the studies in the SR. Primary outcomes were stated for 30/47 studies. The primary outcome reported in the literature differed from that recorded in the registry in 6/30 studies which specified primary outcomes. Secondary outcomes were recorded for 23 studies. Nine studies reported additional secondary outcomes and seven studies did not report all pre-specified secondary outcomes. All clinician-reported outcome measures from the SR were found in the registry review. 12 studies had a quality of life (QoL) or pain endpoint, but only three of these specified an instrument.

## Conclusion

Despite the inconsistencies on a per-trial basis, searching the registry provides a comprehensive overview of clinician-reported outcomes used in this field. However there is a limited range of outcomes used in PC trials. The trial registry search did not yield good results for PRO data. Searching trials registries may provide an alternative to a SR although this should be validated in other disease areas.

